# Owned dog population profile, dog ownership and bite risk determinants: Key steps to rabies control in Antananarivo, Madagascar

**DOI:** 10.1371/journal.pone.0360417

**Published:** 2026-10-16

**Authors:** Marie Hermelienne Volasoa, Santatriniaina Michel Joharison Andrianarivelo, Katerina Albrechtova, Fenitra Raharijaona, Daouda Kassié, Véronique Chevalier

**Affiliations:** 1 International Centre of Research in Agriculture for Development (CIRAD), UMR ASTRE, Antananarivo, Madagascar; 2 Department of Veterinary Medicine and Science Education (DESMV), University of Antananarivo, Antananarivo, Madagascar; 3 Epidemiology and Clinical Research Unit, Institut Pasteur de Madagascar, Antananarivo, Madagascar; 4 International Centre of Research in Agriculture for Development (CIRAD), UMR ASTRE, Montpellier, France; Indira Gandhi Medical College and Hospital, INDIA

## Abstract

Rabies remains a significant public health threat in Madagascar, with dog-mediated transmission causing approximately 1,000 human deaths annually. In Antananarivo, the capital city, control efforts are hindered by limited up to date knowledge on the dog population. This study aims to provide updated estimates of the owned dog population characteristics, identify determinants of ownership, and assess dog bite incidence and risk factors to guide effective vaccination and prevention strategies. Between 2023 and 2025, a longitudinal door‑to‑door household survey was conducted across 21 *fokontany* (administrative units) in Antananarivo. Of 2,518 surveyed households, 30.5% reported dog ownership, with 1,273 dogs recorded, yielding an estimated dog‑to‑human ratio of 1:9. The owned dog population was predominantly male (63.6%), with a median age of three years. Survival analysis in the selected study site indicated a median lifespan of 4.4 years and an annual mortality hazard of 16%. Vaccination coverage was low: 64.2% of owned dogs had never been vaccinated, primarily due to owners’ time constraints, lack of awareness about the need for dog rabies vaccination, and financial barriers. Ownership was positively associated with household location and size, household head’s age, education and income. No significant difference in bite risk was observed between dog‑owning and non‑dog‑owning households, whereas households with children under 15 years experienced a significantly higher risk. Rabies elimination among the owned dog population in Antananarivo requires sustained, context‑adapted interventions that address high population turnover. Annual city‑wide dog vaccination campaigns combining door‑to‑door strategies with fixed‑point sites are suggested to increase the chances of reaching a larger share of the owned dog population. Public health efforts must also prioritize paediatric bite prevention and expand access to post‑exposure prophylaxis to contribute to the global goal of eliminating dog‑mediated human rabies by 2030.

## Introduction

Rabies remains a persistent public-health threat in endemic countries, with dog-mediated transmission as the principal route to human infection. Despite being preventable, canine rabies still causes thousands of human deaths each year, predominantly in low- and middle-income countries (LMICs) [1]. The most effective control measure is mass dog vaccination (MDV). World Health Organization (WHO) guidance recommends vaccinating at least 70% of the total dog population to substantially reduce and ultimately interrupt dog-to-dog transmission and prevent transmission to humans [2]. Dog demography and the nature of human-dog relationships determine the success and durability of vaccination campaigns [3]. Dog demographic processes such as birth and death rates shape population size and density and therefore the proportion of immune animals in a given population [4,5]. When the dog population has a short life expectancy, vaccination coverage can fall by 30–50% within a year after reaching the 70% target, undermining campaign effectiveness [6]. Ownership patterns further influence vaccination outcomes: owned dogs with poor healthcare that roam freely are more susceptible to infection and more likely to contribute to onward transmission [7]. Furthermore, because free-roaming dogs are often difficult to vaccinate, they can sustain rabies transmission cycles within the canine population [8]. Collectively, these factors reduce vaccination coverage and weaken the population-level impact of immunization. Thus, effective and sustainable vaccination programs require a clear assessment of dog population profiles and human–dog relationships.

Canine rabies is a high-priority zoonosis in Madagascar, causing an estimated 1,000 human deaths annually [9]. National policy historically relied on dog culling under Decree No. 95−375 (1995), but the government has shifted toward MDV in line with WHO guidance [9,10]. The first national MDV campaign was launched in 2019 by the Ministry of Agriculture and Animal Health (MAAH) with GAVI support, but the COVID-19 pandemic disrupted the effort which resulted in low vaccination coverage [11]. Later, the national rabies control plan 2022–2027 was launched, with MDV as one of its priorities [10], yet operational constraints of the theoretical document mirror those seen across many LMICs. A critical barrier is the lack of reliable, up-to-date data on dog population size, demographic structure, and human-dog interactions. Madagascar lacks a central registry of owned dogs; many owned animals roam freely and mix with ownerless dogs, and ownerless dogs are not systematically monitored. This absence of data leaves vaccination coverage uncertain and jeopardizes effective vaccination program implementation.

In rabies-endemic areas, every dog bite should be treated as a potential exposure and every bite victim should promptly seek post-exposure prophylaxis (PEP) to prevent a fatal outcome. Yet, many bite victims do not receive this lifesaving treatment because of limited awareness, financial barriers, poor access to healthcare, and other social or logistical obstacles [12]. To improve PEP access in Madagascar 32 PEP centres have been progressively established across the country’s districts, with each centre serving on average 700,000 people [13]. Nationwide, approximately 15,000 bite victims seek PEP annually, with the capital city, Antananarivo, accounting for 60% of these patients. For instance, in 2024 alone, Antananarivo reported 6,731 bite cases [14]. These figures, however, likely underestimate the true burden, as active bite surveillance is lacking. Therefore, identifying risk factors for human bites is essential to pinpoint vulnerable populations and guide targeted interventions that improve healthcare access, strengthen community engagement, and raise awareness to prevent dog bites.

As the site of the Rabies National Reference Laboratory, Antananarivo reports the majority of declared rabies cases. Consequently, the capital has become the focal point of rabies control programs, particularly through dog vaccination and population management. Nevertheless, cases continue to occur, with owned dogs constituting 87.4% of reported rabies incidents [11]. The most recent dog ecology study conducted in Antananarivo dates back more than 15 years, when dog density was estimated at one dog per 4.5 inhabitants with 88.7% of dogs classified as owned [15]. Since then, the capital has undergone substantial demographic and urban changes; however, rabies control strategies continue to be informed by figures from that earlier period. This reliance on obsolete estimates creates a critical gap between current epidemiological realities and the evidence base guiding intervention efforts.

This study proposes updated data on the owned dog population and bite incidence in Antananarivo, using a door-to-door household survey. The objectives are to: (i) estimate the abundance, the demographic characteristics and parameters of the owned dog population in Antananarivo, (ii) identify household-level determinants of dog ownership, including socioeconomic factors that influence both the decision to own a dog and the dog’s roaming behaviour, and (iii) estimate human bite incidence and associated risk factors.

## Methods

### Study area

The study was carried out in 2023–2025 in the Urban Commune of Antananarivo (CUA), the capital of Madagascar. The CUA covers 86.5 km^2^ and includes dense residential neighbourhoods, peri-urban expansion areas, and nearby rice fields. The estimated human population in early 2025 was 1,391,433 [16]. Administratively, the CUA is divided into six Arrondissements, which together contain 192 *fokontany*, the smallest administrative units. Based on expert advice from the CUA and an assessment by the Bureau National de Gestion des Risques et Catastrophes (BNGRC) [17], 21 *fokontany* were selected (Fig 1) for the study based on socioeconomic, safety, and accessibility criteria, with an even spread across the six Arrondissements. Socioeconomic classification was defined by the average proportion of high- and low-income households within each *fokontany*. Eighteen economically vulnerable *fokontany* (≥60% low-income households) were chosen first, on the assumption that their populations might be more affected by rabies. To increase variability, three economically affluent *fokontany* (≥60% high-income households) were later included. Together, the selected *fokontany* cover about one-fifth (20%) of the CUA surface area (calculated using QGIS 3.4.2).

**Fig 1:**
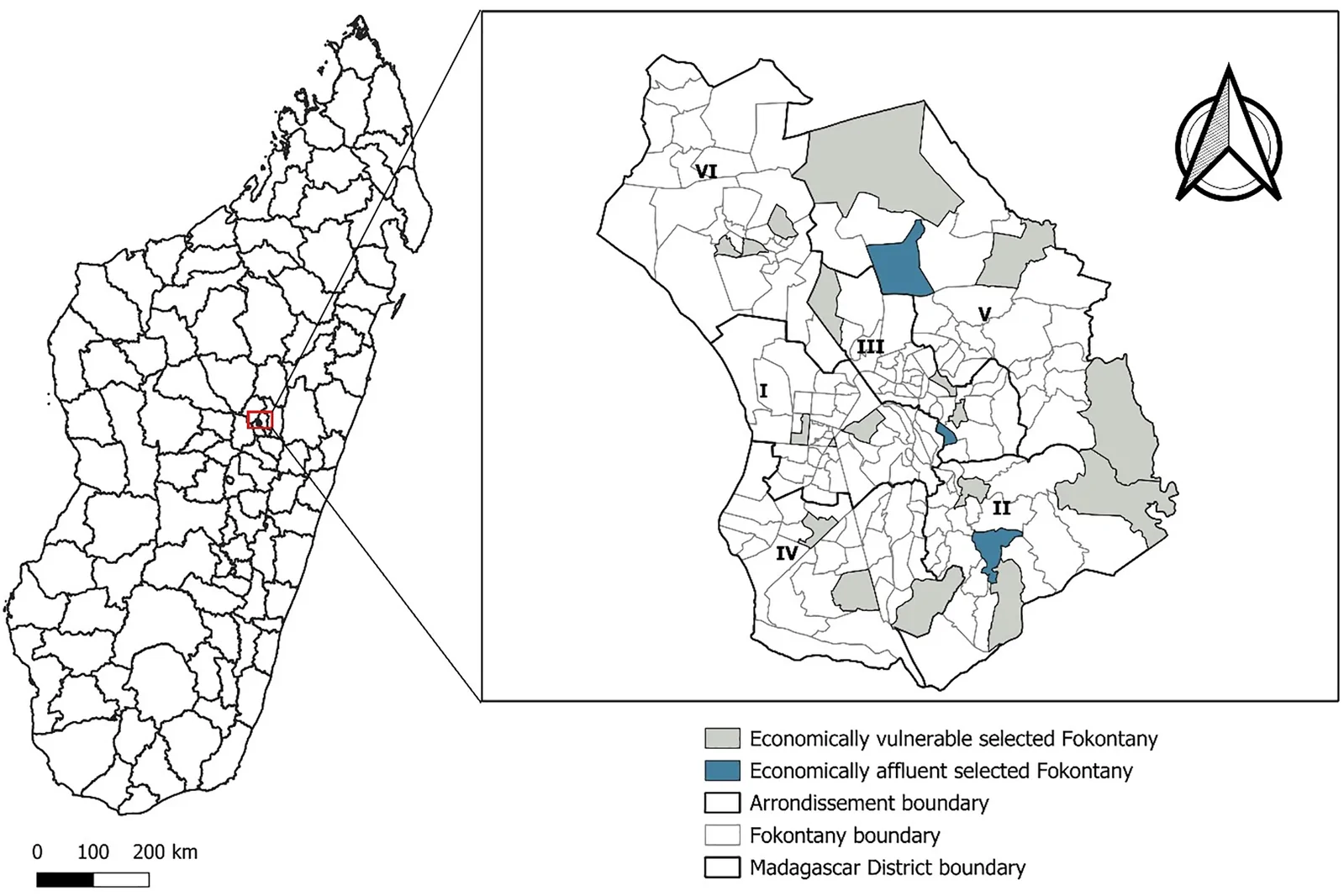
Location of selected *Fokontany* within the Urban Commune of Antananarivo, Madagascar 2023-2025 (n = 21). Economically vulnerable and affluent *fokontany* are shaded in grey and blue, respectively. Map production were performed using QGIS version 3.18.3. Administrative boundaries source: BNGRC/OCHA (Humanitarian Data Exchange), CC BY-IGO, available at: https://data.humdata.org/dataset/cod-ab-mdg.

### Census and survey

Household surveys were undertaken in the selected *fokontany* using a biphasic door-to-door transect method. Within each *fokontany*, an initial target sample of at least 100 households was established. This threshold corresponded to approximately 500 individuals per *fokontany*, based on an average household size of five persons according to official census data. Assuming an expected dog bite incidence of 0.5%, an Intraclass Correlation Coefficient of 0.02, and a precision of 0.5%, 30 households (≈150 individuals) per *fokontany* is required. A larger sample of 100 households was chosen to reduce the design effect, accommodate high between cluster variability, and remain within field and financial capacities. The larger sample also comfortably integrates the anticipated 10% non-response rate, ensuring that the final sample remains robust after accounting for refusals or absences. On average, the 100-household target represented 4.4% of households per *fokontany*.

All interviews were done in Malagasy by trained interviewers to ensure participants understood the questions and to reduce information bias. Data were collected with Kobo Collect (https://www.kobotoolbox.org) and stored centrally on Kobo Toolbox for real-time monitoring.

Prior to fieldwork, preliminary assessments validated the proposed study sites through direct observations and consultations with *fokontany* leaders. These steps helped plan the logistics, improve survey implementation, and engage the community.

#### Baseline household survey and dog census (S1).

Between November 2023 and February 2024, structured household (household being defined as a group of individuals who live within the same residence and share socioeconomic needs associated with production and consumption) [18] interviews (S1) were conducted in the 18 vulnerable *fokontany*, and in February 2025, in the 3 economically affluent *fokontany*. Interviews targeted household heads and, when applicable, individuals who had experienced dog bites the year before the interview. Three standardized questionnaires were used: (i) Household questionnaire capturing socio-demographic information (household head’s age, gender, income, and education level), number of household members (household size, and the number of children under 15 years. (ii) Dog questionnaire, recording the number of dogs owned by the family, their age, breed, sex, origin (gift, purchase or found), roaming behaviour (confined indoors with no outdoor access, partially confined with occasional outdoor access, or free-roaming with unrestricted outdoor access), reproductive status (neutered or not, pregnant or not if female), number of litters of each female, the fate of pups (kept, sold, given away or killed), the role of dogs within household (security purpose, companionship, or as a source of income) and rabies vaccination status (has received at least one vaccine dose in their lifetime, currently up-to-date on vaccination, never vaccinated). When owners reported that a dog had been vaccinated, interviewers asked to see the dog’s vaccination card and cross-checked the reported vaccination status against the available documentation whenever possible. When the vaccination card was unavailable or could not be produced at the time of the interview, vaccination status was based on the owner self-report. Each identified dog had been assigned a unique identifier, with detailed characteristics (name, sex, age, breed) and was photographed (front and profile) to allow follow-up tracking. (iii) Bite victim questionnaire collected socio-demographic information (age and gender of the victim), and the incident location (in the household, in the neighbourhood street, in another neighbourhood), a description of the biting animal (breed, sex, age, and ownership status, if applicable).

#### Follow-up survey (S2): dog population dynamic.

Within the 18 economically vulnerable selected *fokontany*, a follow-up survey (S2) was conducted eight months later (August–September 2024). Only households that reported dog ownership during S1 were revisited. This phase focused on tracking previously recorded dogs, identifying dogs no longer present in the household, and documenting the reasons for their absence. Each dog encountered during S2 was vaccinated against rabies using subcutaneous injection of Rabisin ® [19] (supplier: Merial, Madagascar).

For all households successfully re-interviewed during S2, owners were asked to confirm dog identities using S1 photographs. A dog recorded during S1 was classified as “still present” if it was directly observed by the survey team or reported alive by the owner, even when not seen during the visit. Dogs no longer present were classified into two categories: (i)“dead,” when the owner reported that the dog had died, specifying the cause (disease, road accident, poisoning, or old age) and the date of death; (ii) and “lost to follow-up”, when the dog had either moved to another household or disappeared, with its subsequent outcome unknown, along with the date it was last seen in the household.

### Statistical analysis

Data were downloaded from the Kobo Toolbox server as comma-separated value (CSV) files, cleaned, and analysed using R version 4.5.1 (R Core Team 2025, https://www.R-project.org/). Statistical significance was defined as a two-sided p-value < 0.05.

The dog-to-human and male-to-female ratios were calculated respectively, as the total number of dogs recorded over the sum of participant household sizes, and sum of total males recorded over the sum of total females. The variations of these characteristics across *fokontany* were analysed using χ^2^ tests.

#### Dog demography.

Dog age distribution was summarized using mean and median ages and compared across fokontany using the Kruskal-Wallis test, followed by pairwise Wilcoxon tests for post-hoc comparisons between fokontany. The cumulative age distribution was described using the empirical cumulative distribution function (ECDF) [20]. Reproductive characteristics were assessed by calculating the proportion of sterilized dogs, the number of females of reproductive age (≥ 8 months [21], the proportion of females that had given birth in the preceding six months, the median litter size, and the ratio of reproductive females to puppies. Kaplan-Meier survival estimates were generated using data from dog-owning households in the 18 economically vulnerable fokontany [22]. For this analysis, only households that were successfully re-interviewed during the S2 were included, as they provided the only reliable longitudinal data from the initial census (S1). The follow-up period for each dog was defined as the time interval between the first interview (S1) and either the recorded date of death or loss to follow-up, or the date of the second interview (S2) for dogs still alive at that time. Survival analysis was performed using the R survival package; the so-called “extended KM survival algorithm” was used handling the left truncation and right censoring of the data [22–24]. Kaplan-Meier curves with age as timeline were constructed to visualize the survival distributions using the ***ggsurvfit*** and ***survminer*** packages. Dogs reported as deceased were coded as event (event = 1), whereas dogs still alive at S2 or lost to follow-up were censored (event = 0). Loss to follow-up was considered when the dog was reported missing since a given date between S1 and S2 and the owner was not sure whether his animal died after that date. We assumed non-informative right censoring, i.e., that, conditional on the information available at the time of censoring, subsequent survival was independent of the probability of being lost to follow-up. The log-rank test was used to assess any difference of survival rates between sex.

Survival estimates were used to assess the decline in vaccination coverage following a hypothetical single dose MDV campaign achieving 70% coverage of the owned dog population. The annual mortality hazard (*h*) was derived from the median survival time assuming an exponential survival model, where *h* equals the natural logarithm of 2 divided by the median survival time (*h* = *ln* (2)/ T*median*), which provides a parsimonious representation of overall dog turnover. A constant mortality hazard, identical across age classes and vaccination status, was assumed to avoid introducing additional uncertainty related to age structure or differential survival. The dog population was further assumed to be demographically stable, allowing changes in vaccination coverage to be attributed solely to mortality. Finally, post-vaccination immunity was assumed to remain constant over the period considered, consistent with previous modelling approaches. These simplifying assumptions were adopted to isolate the effect of dog turnover on vaccination coverage dynamics.

#### Dog ownership.

To consider overdispersion, ownership factors were analysed using a negative binomial regression model, with the number of dogs per family as model outcome. The explanatory variables were: the number of children under the age of 15 in the household; the gender of the household head (male or female); the age of the household head, categorized into five groups (<30, 30–40, 40–50, 50–60, and >60 years); the household head’s monthly income, classified relative to the Malagasy minimum wage [25] (SMIC, 250,000 Ariary or approximately 55 USD) as <250.000 Ariary (<55 USD), 250.000–500.000 Ariary (55–110 USD), and >500.000 Ariary (>110 USD); the education level of the household head, categorized as no formal education, primary education, lower secondary education, upper secondary education, or university level.; the number of household members and the household’s Arrondissement of residence (1–6). Collinearity among the variables was assessed using the Variance Inflation Factor (VIF); variables with VIF < 5 were retained for inclusion in the baseline model. The final model was selected using a stepwise forward and backward selection procedure based on the Akaike Information Criterion (AIC) [26].

#### Bite incidence and risk factors.

Bite incidents were described in terms of their frequency, temporal distribution, characteristics of biting animals (demographics and ownership status), and circumstances of the events. Temporal trends were examined by aggregating bite cases by month of declaration and visualizing their monthly distribution. The risk factors for bite incidents were evaluated using two complementary approaches. First, descriptive analyses were conducted to characterize the profile of bite victims according to age, sex, education level, income, and relationship to the dog. Differences in proportions across categories were assessed using Chi-square (χ^2^) tests. Second, risk factors at the household level were identified using a Generalized Linear Model (GLM) with a binary outcome indicating the occurrence of at least one bite incident in the household. Explanatory variables included the number of children under 15 years of age in the household; the sex, age, income, and education level of the household head; the number of owned dogs; and the Arrondissement of residence. Model selection was carried out using a stepwise forward and backward stepwise procedure based on the AIC.

## Ethical consideration

The study protocol was reviewed and approved by the Urban Commune of Antananarivo, where the research was conducted, and the Direction of Veterinary Services (DSV) under the Ministry of Livestock. The National Ethics Committee for Biomedical Research waived the requirement for ethical review. Prior to each interview, participants received detailed explanations regarding the study objectives, procedures, and potential implications. Written informed consent was obtained from all participants, who were informed of their right to decline or withdraw at any time without consequence. Written consent was also obtained from dog owners for animal vaccination and photographic records. Personal identifiers were used only during the S2 phase to allow follow-up and were then removed prior to statistical analysis to ensure full anonymization. All dog vaccinations in S2 were conducted under the authority and supervision of the DVS, Madagascar.

## Results

### General characteristics of surveyed households and dogs

Across the 21 selected *fokontany*, a total of 2,518 households were visited, representing 11,150 individuals. Household sizes ranged from 1 to 20 occupants, with a median of 4 persons. The average age of the respondents was 43 years (*sd* = 15). Women represented 66.8% of the respondents, and 47.5% of interviewed participants were household heads. A total of 1,273 dogs were recorded during phase S1, distributed across 767 households, accounting for 30.5% (95% CI: 28.6–32.3) of the sampled households. On average, each dog-owning household had 1.6 dogs (*sd* = 1.2, min = 0, max = 14). General information on censused dogs is provided in S1.1 Table. The estimated overall dog-to-human ratio was 1:9, exhibiting significant variability across *fokontany* (p < 0.001), with values ranging from 1:5–1:16. When regrouped according to the prevailing socioeconomic class in the *fokontany*, this ratio varied slightly by socioeconomic context, with dog-to-human ratios of 1:8.6 in economically vulnerable *fokontany* and 1:9.3 in economically affluent *fokontany*. Aggressive behaviour was noted in 232 dogs (18.2%, 95% CI: 16.2–20.4). Most dogs were of the local breed (870/1,273; 68.5%, 95%CI: 65.9–71.2), with exotic breeds making up 31.3% (95% CI: 28.8–34.1; 399/1,273 dogs). Breed information was missing for 4 dogs (0.2%).

### Dog demography

#### Sex ratio, age distribution and reproductive profile of the studied dog population.

Among the 1,273 recorded dogs, males accounted for 63.6% (809/1,273) and females 36.4% (464/1,273), resulting in an overall female-to-male ratio of 1:2. This ratio varied significantly across *fokontany* ranging from 1:0.4 to 1:2 (p < 0.001). Stratified analyses revealed marked differences by *fokontany* socioeconomic status. In the 18 economically vulnerable *fokontany*, the female-to-male ratio was estimated at 1:2, with substantial variation across *fokontany* (ranging from 1:0.5 to 1:30, p < 0.001). In contrast, the three economically affluent *fokontany* exhibited a more balanced ratio of 1: 1.5, with limited variability (range: 1:1.3–1:1.7) and no statistically significant differences across *fokontany* (p > 0.05).

Of the 1,273 censused dogs, 215 (16.9%) were classified as puppies (0–6 months), 195 (15.3%) as subadults (6–12 months), 641 (50.4%) as adults (1–5 years), and 222 (17.4%) as older dogs (>5 years). Fig 2 illustrates the cumulative age distribution of the owned dog population. The estimated median age was 3 years (~36 months), and the oldest recorded dog was 16 years. Age distributions differed significantly across *fokontany* (Kruskal-Wallis test, p < 0.001), with median ages ranging from 1.5 to 4 years. Pairwise Wilcoxon rank-sum tests indicated that dogs from several economically vulnerable *fokontany* in Arrondissement 4 and 6 (Namontana, Anosibe, and Antanety Atsimo) were younger than the overall study population (p < 0.05), whereas dogs from some economically affluent *fokontany* in Arrondissement 2 and 3 (Ambohimiandra and Ankadivato) tended to be older (p < 0.05) (S1.2 Table). The vast majority of dogs were not sterilized (1,218/1,273; 95.7%), with only 55 (4.3%) having been spayed or neutered. Among the 467 female dogs recorded, 385 (82.4%) were of reproductive age (≥8 months). Of these, 43 (11.2%) had given birth in the six months preceding the survey, with a median litter size of five puppies. This corresponds to an estimated reproductive output of 0.6 puppies per female of reproductive age per cycle.

**Fig 2:**
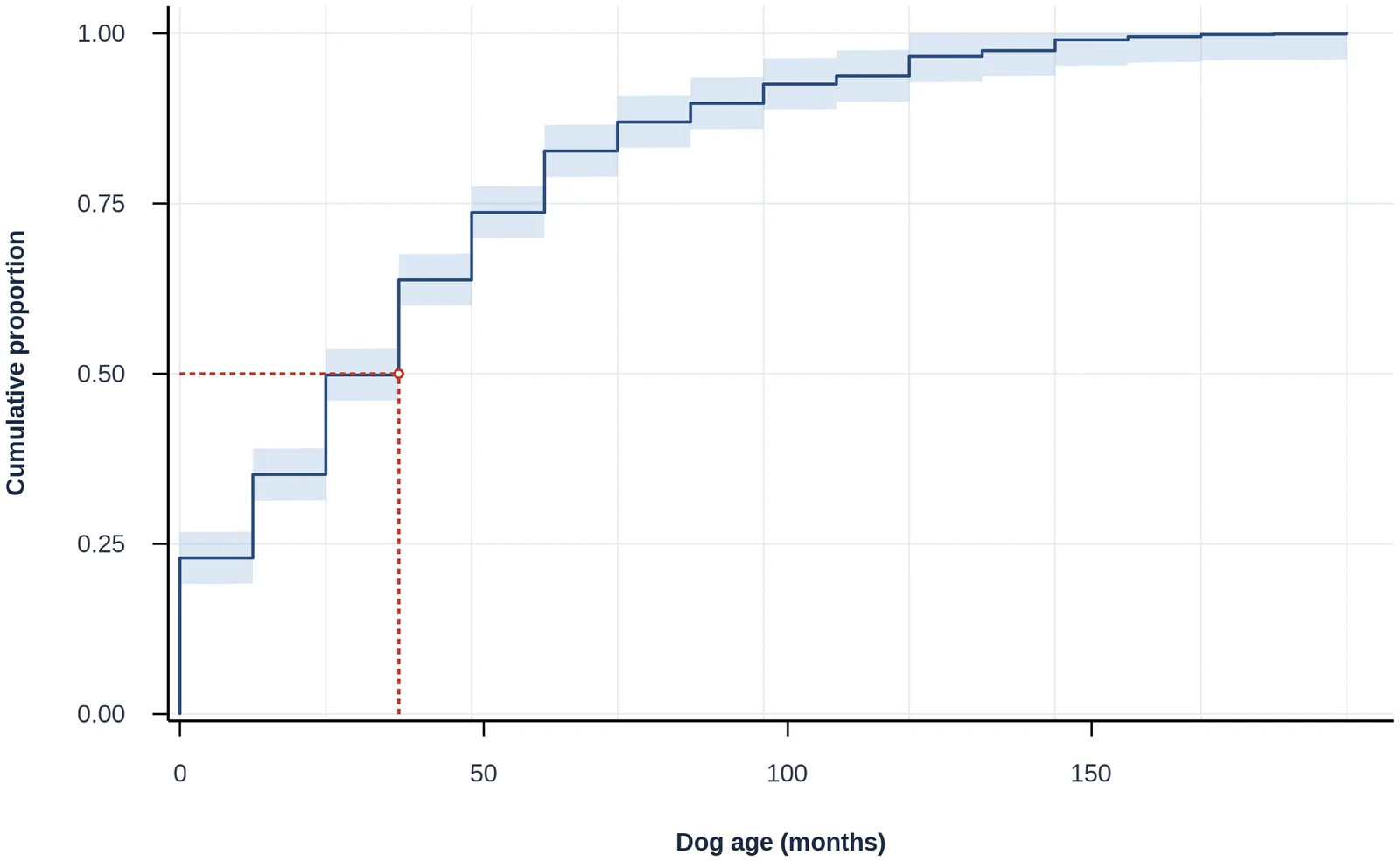
Cumulative age distribution of the owned-dog population in the Urban Commune of Antananarivo, Madagascar 2023–2025 (n =  1,273). Empirical cumulative distribution function (ECDF) of dog age (in years) among the censused owned population. The horizontal red dashed line represents the 50% cumulative proportion, and the vertical red dashed line marks the estimated median age of the dog population.

#### Survival analysis.

During the initial survey (S1) conducted across 18 *fokontany*, a total of 1,121 dogs were recorded. At the follow-up survey (S2), 937 dogs (83.6%) were successfully re-identified. Of these, 120 had died and 54 were reported absent, while the remainder were confirmed alive at the time of revisit. The 16.4% left (n = 184 dogs) could not be assessed due to the absence of a household respondent during S2; they were not included in the survival analysis. Details of the follow-up status of the dog population are provided in S2 Table. Kaplan–Meier survival estimates (Fig 3) showed a progressive decline in survival probability with increasing age, with a marked decrease during the first year of life. Approximately 70.0% (95% CI: 0.7–0.8) of dogs survived beyond one year of age. Conditional on survival to the age at first observation, the estimated median survival age was 4.4 years (~53 months, 95% CI: 49.5–73.0 months), after which the survival curve steepened. Survival beyond 10 years of age was uncommon, with only a small proportion of dogs reaching 15 years. Overall survival did not differ significantly between males and females.

**Fig 3:**
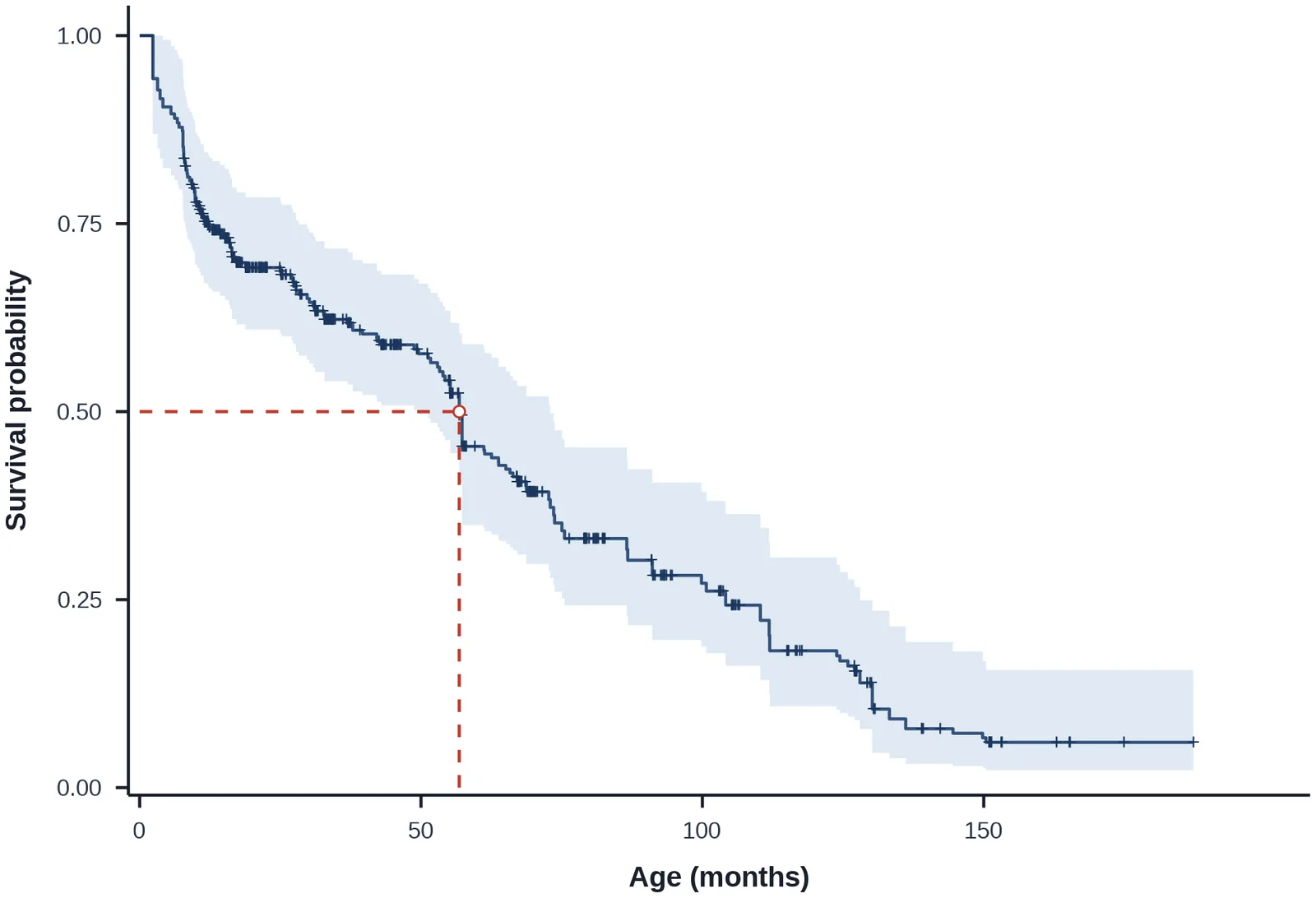
Survival curve of the owned dog population in the Urban Commune of Antananarivo, Madagascar (2023-2024, n = 937). Kaplan–Meier estimate of dog survival by age (months). Dogs entered the analysis at their age at the initial survey (left truncation) and were followed until death or censoring due to loss to follow-up or end of follow-up (8 months). The solid line shows the survival function, the shaded area, the 95% confidence interval, and tick marks censored observations. Dashed lines indicate the median survival age.

Under the assumptions described in the Methods section, a median lifespan of 4.4 years corresponds to an annual mortality hazard of approximately 0.16 (16.0% per year). Accordingly, following a hypothetical single vaccination campaign achieving 70% coverage among owned dog populations, the vaccination coverage would be expected to decline to approximately 60% after one year due to mortality alone.

### Dog ownership

#### Motivation for dog acquisition.

The primary reason for dog ownership was security purposes (dogs used as guards of house and family possessions), with 65.0% (95% CI: 0.6–0.7) of households citing this as their main motivation. Dogs were obtained from various sources. The most frequent was receiving a dog as a gift (31.3%), followed by purchase (25.4%), birth within the household (25.1%), and being found on the street (18.3%). More than half of owners (54.9%, 95% CI: 51.1–58.6) preferred males, while 37.9% (95% CI: 34.3–41.6) had no preference, and only 7.1% (95% CI: 5.1–8.9) preferred females.

#### Management practices.

Regarding offspring management, 37.4% of dog owning households gave puppies to acquaintances, while a similar proportion (37.0%) chose to keep them. Additionally, 16.0% reported abandoning puppies, and 8.6% sold them (generally as an occasional source of income rather than a regular one). Euthanasia of unwanted puppies was reported by 1.0% of households. In terms of nutrition, the vast majority of households (96.8%) across both economically vulnerable and affluent *fokontany* fed their dog(s) at least once per day. Rabies vaccination coverage was low: 64.2% (CI 95%: 61.5–66.8) of dogs had never received any vaccines. The main reasons for not vaccinating were lack of time, reported by 30.1% of households (95% CI: 25.4–34.8), lack of awareness (26.6%, 95% CI: 22.0–31.1), i.e., owners knowing that dog vaccination is available but lack a clear understanding of its needs to be done in context of endemic country as Madagascar, and financial constraints (26.6%, 95% CI: 22.0–31.1). Additionally, 16.7% (95% CI: 12.9–20.5) of households cited other reasons, such as indifference to/or opposition against vaccination. Regarding roaming behaviour, 56.8% (95% CI: 53.1–58.1) of dogs were left to roam free, either continuously or intermittently.

#### Determinants of household dog ownership.

According to the AIC, the final model retained household location (Arrondissement level), the age and gender of the household head, their education level and monthly income, as well as the total number of household members and the number of children as explanatory variables. The negative binomial regression model revealed distinct household-level drivers of dog ownership (Table 1). Geographically, households in the peripheral Arrondissements (3–6) owned significantly more dogs than those in the city centre (Arrondissement 1, Reference), with Incidence Rate Ratios (IRR) ranging from 1.8 to 2.1 (p < 0.001). Arrondissement 2, however, did not differ significantly from the reference area (IRR 1.0, p > 0.05). Demographic factors also played a central role. Dog ownership rates were significantly higher when household heads were more than 30 years old compared to those under 30 (Reference), with IRRs between 1.6 and 2.2 (p < 0.05). Household size showed a similar positive trend, each additional member increased the expected number of dogs by 7% (IRR 1.1, 95% CI: 1.0–1.1, p < 0.05). Regarding socioeconomic status, higher income was associated with an increased number of owned dogs: households earning >500,000 Ar (> $110) had 39.0% more dogs than the lowest income group (<250,000 Ar / < $55, Reference) (IRR 1.4, 95% CI: 1.1–1.8, p = 0.01). Conversely, education showed an inverse relationship: household heads with primary education owned significantly fewer dogs than those with no formal education (Reference) (IRR 0.56, 95% CI: 0.3–0.9, p < 0.05). Neither the sex of the household head nor the number of children under 15 years showed a significant association with dog ownership.

**Table 1:** Household-level determinants of dog-ownership in the Urban Commune of Antananarivo, Madagascar, 2023.

		Estimate	Incidence rate ratio (CI 2.5%−97.5%)	p- value
	Intercept	−2.04	0.1 (0.07–0.23)	< 0.001
**Arrondissement**	**1**	**Ref**	**Ref**	**Ref**
	2	0.01	1.0 (0.7–1.4)	0.9
	3	0.61	1.8 (1.4–2.4)	< 0.001
	4	0.7	2.0 (1.5–2.7)	< 0.001
	5	0.6	2.0 (1.5–2.5)	< 0.001
	6	0.7	2.1 (1.6–2.8)	< 0.001
**Household head age in year**	**30**	**Ref**	**Ref**	**Ref**
	30-40	0.5	2.2 (1.5–3.1)	< 0.05
	40-50	0.7	1.6 (1.1–2.3)	< 0.001
	50-60	0.7	2.0 (1.4–2.8)	< 0.001
	>60	0.8	2.1 (1.4–3.0)	< 0.001
**Sex of household head**	**Women**	**Ref**	**Ref**	**Ref**
	Man	0.1	1.2 (0.9–1.4)	0.1
**Head of household monthly income**	< 250 000 Ar (<$55)	**Ref**	**Ref**	**Ref**
	250 000–500 000 Ar ($55 - $110)	0.05	1.0 (0.8–1.3)	0.6
	> 500 000 Ar (> $110)	0.3	1.4 (1.1–1.8)	0.01
**Head of household education**	**No formal education**	**Ref**	**Ref**	**Ref**
	Primary education	−0.6	0.7 (0.3–0.9)	0.02
	Secondary education	−0.4	0.80 (0.5–1.3)	0.05
	High school	−0.2	0.6 (0.4–1.0)	0.3
	University	0.0	1.0 (0.6–1.6)	0.9
	**Household size**	**0.07**	**1.0 (1.0–1.1)**	**< 0.05**
	**Children <15 yrs.**	**−0.05**	**0.9 (0.8–1.0)**	**0.2**

Results of a negative binomial regression model assessing factors associated with the number of dogs per household. Explanatory variables included Arrondissement of residence, household head’s age, gender, education level, and monthly income, household size, and number of children under 15 years. Estimates are presented as incidence rate ratios (IRR) with 95% confidence intervals and corresponding p-values.

### Bite incidence and risk factors

In 2023, 59 bite events were recorded among 11,150 household members of survey participants, corresponding to a yearly bite incidence of 0.5% (95% CI: 0.4–0.7). About three quarters of reported bite incidents (42/59) occurred in households without dogs. Bite incidents were reported in 3.2% of dog owning households compared with 4.3% of non-dog owning households, with no significant difference in risk between the two groups (RR = 0.7, 95% CI: 0.4–1.2, p = 0.29). Bites primarily took place on the victim’s neighbourhood street (44.1%), followed by the victims’ home (28.8%), and in other neighbourhoods (27.1%). According to participants, the dogs involved were predominantly owned by other known persons (50.9%, 95% CI: 51–83), followed by stray dogs (30.5%) and dogs owned by the victims themselves (18.6%). Bite victims were mostly males (66.1%, 95% CI: 0.4–0.7, p < 0.05), with a median age of 26 years (min = 2, max = 67). The majority of victims (77.9%) were older than 15 years. A visual analysis of bite incidents by month showed a notable peak in October, although January, June, July, August, and December also recorded relatively high numbers of cases (Fig 4). The final regression model included the Arrondissement and the number of children under 15 years per household as explanatory variables for bite risk (Table 2). Households with more children under 15 years were significantly more likely to report a bite incident (OR=1.2; 95% CI: 1.0–1.4; p < 0.05).

**Table 2:** Household-level risk factors for dog bite incidents, Urban Commune of Antananarivo, Madagascar, 2023.

		Estimate	Odds Ratios (CI 2.5%−97.5%)	p- value
	Intercept	−3.5	0.02 (0.01–0.04)	< 0.001
Arrondissement	**1**	**Ref**	**Ref**	**Ref**
	2	0.06	1.1 (0.5–2.1)	0.8
	3	0.3	1.3 (0.6–2.6)	0.4
	4	0.4	1.5 (0.8–3.0)	0.1
	5	−0.5	0.6 (0.3–1.2)	0.1
	6	−0.7	0.4 (0.17–1.5)	0.08
	**Children <15 yrs**	**0.1**	**1.2 (1.0–1.4)**	**0.03**

Results of a binomial generalized linear model examining factors associated with the occurrence of at least one dog bite per household in 2023. Explanatory variables included Arrondissement of residence and the number of children under 15 years in the household. Estimates are presented as odds ratios (OR) with 95% confidence intervals and p-values.

**Fig 4:**
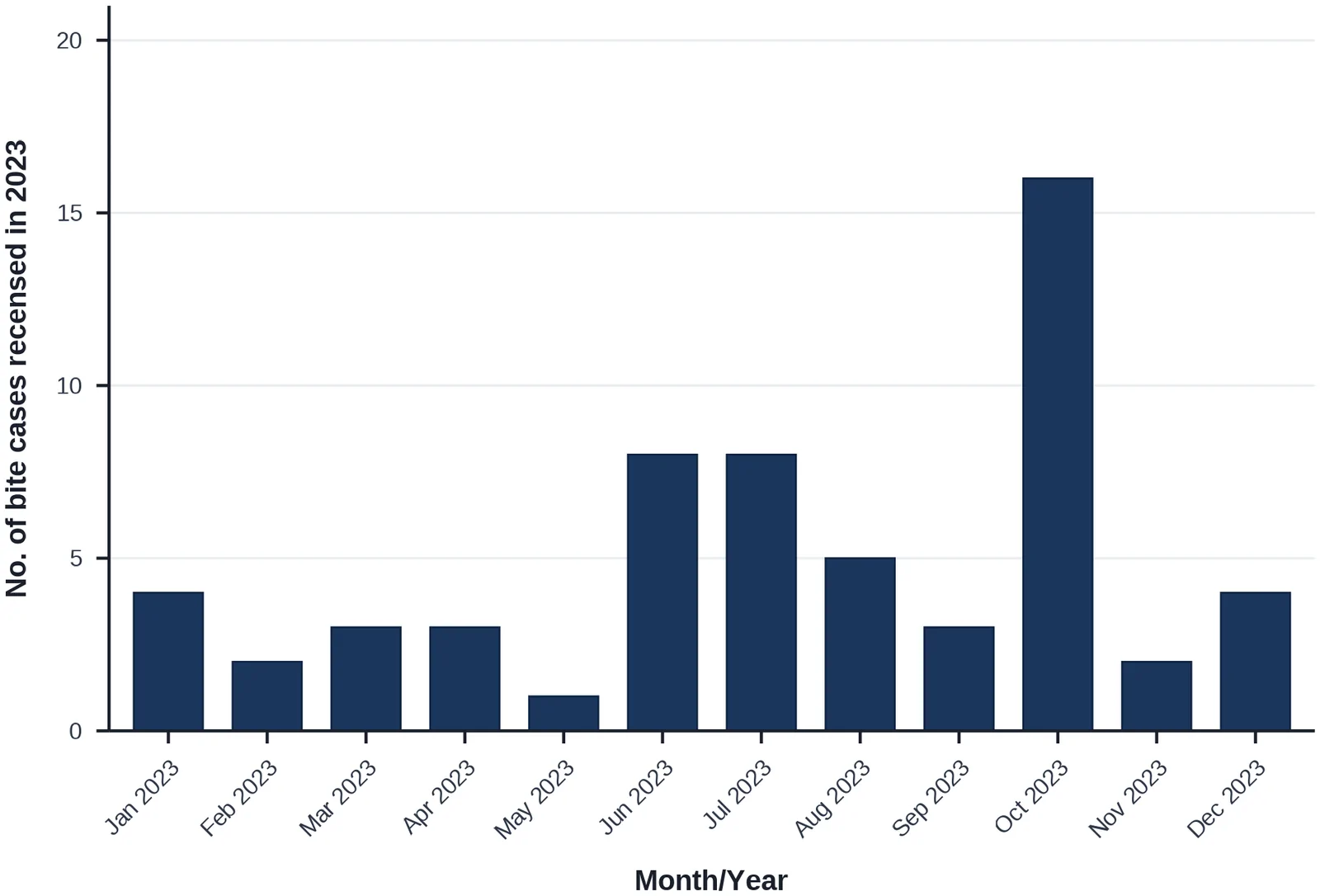
Distribution of declared bite cases per household and by month of occurrence, Urban Commune of Antananarivo, Madagascar, 2023 (n = 59). The absolute number of recorded bite cases is displayed by month.

## Discussion

In Madagascar, rabies remains a serious public health concern, particularly in the CUA, the capital city, where up‑to‑date information on dog ecology is almost non-existent. Our study seeks to bridge this gap by presenting the results of a thorough owned dog population survey. We also took advantage of this fieldwork opportunity to document bite incidents and related circumstances, providing additional insight into rabies exposure in a country that relies heavily on passive surveillance [10]. We selected 21 *fokontany* (10.9% of all *fokontany* in the capital), classified as economically affluent or vulnerable based on the average proportion of low‑ and high‑income households, to conduct a dog census and survey dog ownership and bite cases. In vulnerable areas, long-term data was collected to estimate dog population dynamic parameters, as these directly affect vaccination immunity coverage [27]. We acknowledge that the non-random selection of study sites may constitutes a methodological limitation and may limit the generalizability of our findings to the wider CUA.

In our survey, 30.5% of urban households reported owning at least one dog, corresponding to an overall dog-to-human ratio of approximately 1:9. When aggregated by socio-economic classification, dog density was higher in economically vulnerable *fokontany* (1:8.6) than in affluent areas (1:9.3), but the difference was not statistically significant. In contrast, the ratio varied substantially across *fokontany* (range 1:3–1:15), suggesting that local heterogeneity is a stronger driver of variation than socio economic status. Note that, given the purposive, vulnerability-based selection of study sites, our estimates should be interpreted with caution. However, these results contrast sharply with the results of a previous study conducted in 2007 in Antananarivo which reported substantially higher household dog ownership (88.9%) and a dog-to-human ratio of approximately 1:4.5 [15], as well as with a more recent study in 2016 from northwest Madagascar, in Mahajanga district, documenting 85% household ownership [28]. Such differences may reflect, on a national level, a profound change in urban living conditions over time. Increased awareness of rabies and associated risks, together with rapid urban population growth, estimated at 4.5% annually [16], may further constrain dog ownership by reducing available space and increased perceived costs of keeping dogs [29]. If confirmed by further longitudinal data, this apparent decline in dog ownership in CUA has important implications for rabies control. Lower dog-to-human ratios mean fewer owned dogs would need to be vaccinated to reach the WHO recommended 70% coverage [30], potentially easing logistical and financial demands of MDV. However, a declining ownership ratio may not reflect a smaller overall dog population, but rather a shift toward unowned or loosely owned dogs. Accurate estimation of both owned and ownerless subpopulations therefore remains critical for planning and evaluating vaccination strategies, underscoring the need for further data specifically on the ownerless dog population.

Comparisons with other African urban and national settings further highlight the extreme heterogeneity of dog ownership patterns. Documented dog-to-human ratios vary significantly, ranging from 1:3.3 in Kenya [31], 1:5.4 in Namibia [32], 1:7.7 in South Africa [33], to 1:21.4 in N’Djamena, Chad [34], 1:37.7 in the Democratic Republic of Congo [35], and an estimate of 1:47 at the national level in Uganda [36]. At the African inter-country level, cultural factors including religious norms may partly explain these differences. Predominantly Christian settings such as Madagascar often report higher dog ownership, whereas regions with larger Muslim populations tend to have lower ownership rates. Together, these findings underscore the need for context-specific dog population assessments to guide the design of effective and efficient rabies vaccination campaigns.

Our study highlights important findings on owned dog population characteristics and ownership. For this analysis, dogs were treated as independent observations, although co‑resident animals may share environmental exposures and management practices. The average number of dogs per household was 1.6 per dog‑owning household, with only one household keeping 14 dogs, which disproportionately influenced the mean. Given the generally small household dog numbers, substantial clustering is unlikely, though some residual within‑household correlation cannot be excluded.

Analysis of owned dog age suggests that dogs tend to survive longer on average in affluent *fokontany* than in the economically vulnerable ones. Survival analysis of the owned dog population in the economically vulnerable *fokontany* of Antananarivo revealed distinct survival probabilities across age classes that could be therefore used as a conservative baseline for vaccination planning purposes. Early-life survival was relatively high, with 90% of puppies surviving past the 3-months, 86% surviving up to 1 year (12 months), and approximately 50% surviving beyond 4.4 years (~53 months). This survival profile is consistent with demographic patterns commonly reported in free-roaming and owned dog populations in LMICs, which are typically characterized by rapid population turnover [37]. Such demographic dynamics pose a major challenge for rabies elimination, since the constant introduction of susceptible puppies and the loss of vaccinated dogs through mortality gradually undermine the herd immunity achieved by vaccination campaigns. Despite this rapid turnover, the median survival rate observed in the economically vulnerable *fokontany* of Antananarivo (4 years) appears comparatively higher than in other urban settings. For instance, one of the longest reported median survival rates in urban dog populations is approximately five years in Chile [38], whereas substantial shorter survival rate (around two years) has been reported in many urban settings across Asia and Africa, including Tanzania (1.8 years) [4], Kenya (2.0 years for female dog) [39] and in India (2.8 years) [40]. Relative to these settings, the age structure in Antananarivo suggests a dog population dominated by adult individuals, which represents a valuable opportunity for improving and sustaining vaccination coverage [41,42]. Our projection of survival estimates onto the trajectory of vaccination coverage indicates that, following a hypothetical campaign achieving 70% coverage among the owned dog population, vaccination coverage would decline by approximately 10% after one year due to mortality alone. Recently, the Philippines, Mexico, and Zanzibar, sustained MDV campaigns have achieved substantial reductions in rabies incidence or even elimination, despite comparable dog demographic challenges [43,44]. Bayesian modelling of high-turnover dog populations in Cambodia suggests that a targeted strategy focusing on annual primary vaccinations for dogs under one year, combined with a single booster, can efficiently maintain the 70% coverage threshold while significantly reducing the number of doses required [45]. To build on the successes achieved elsewhere, and talking about owned dogs, we recommend implementing at least one city‑wide vaccination campaign annually in Antananarivo, with particular emphasis on vaccinating dogs under one year of age [13,45]. Complementary interventions aimed at mitigating high puppy mortality, such as community engagement and improved dog husbandry practices, could further stabilize the vaccinated proportion of the population and enhance the long-term impact of vaccination efforts.

In our study area, dogs were primarily kept for household security, which was associated with a strong preference for male dogs, and resulted in an estimated male-to-female ratio of 2:1. Households attributed this preference to both behavioural and practical considerations: male dogs were perceived as more aggressive and territorial, and therefore better suited for guarding, while also being considered easier to manage due to the absence of reproductive cycles and associated concerns related to pregnancy and puppy care. Skewed sex ratios favouring male dogs have been widely reported in diverse settings, including Thailand, India, Ecuador, South Africa, Bali, Chile, Tanzania, and the United States [3,4,33,38,40,46]. The predominance of male dogs may partially constrain the growth of the owned dog population, as males do not directly contribute to reproduction, potentially reducing the influx of susceptible puppies. This demographic structure could, in turn, enhance the efficiency and sustainability of vaccination by limiting population turnover. However, 56.8% of owned dogs in our study were allowed to roam freely, raising concerns regarding disease transmission, uncontrolled reproduction, and the potential expansion of free-roaming or stray dog population [8]. Together, these findings underscore the need for integrated rabies control strategies that combine high vaccination coverage with effective management of dog populations to ensure sustainable population control and long-term rabies prevention.

Moreover, our findings highlight the influence of both demographic and geographic factors on dog ownership patterns in Antananarivo. Higher ownership outside Arrondissement 2 suggests that traditional vaccination efforts (parenteral vaccination) targeting owned dogs may be particularly effective in those zones. In contrast, the lower reported ownership in Arrondissement 2 underscores the need to estimate dog population density, including both owned and free‑roaming dogs to design effective interventions. To properly calibrate vaccination campaigns and achieve herd immunity in such areas [42], conventional approaches should be complemented by alternative strategies, such as oral rabies vaccination directed at free‑roaming populations [47]. At the household level, dog ownership was more common in larger households and those headed by older individuals, consistent with observations from Kenya, Tanzania, and Uganda [31,36,48]. Older household heads may benefit from more stable living conditions and financial resources, facilitating long-term dog ownership. Similarly, in larger households, responsibilities like feeding and supervising dogs can be shared, making dog ownership more feasible [49]. Despite these identifiable ownership patterns, rabies vaccination coverage among owned dogs remained low, with only 35.8% reported as vaccinated. This estimate aligns with reports from other Malagasy cities, including Mahajanga (35.0% coverage) [28], and mirrors suboptimal coverage levels reported elsewhere in Africa, such as Kenya (40.0%) [31]. The principal barriers to vaccination in our study were time constraints, lack of awareness (specifically awareness of the need to vaccinate and its schedule), and financial limitations, each reported by approximately one-quarter to one-third of respondents. These findings indicate that, despite ongoing MDV campaigns led by the MAAH in partnership with several non-governmental organizations (NGOs) [50], a substantial portion of the target population remains unreached. It should be noted, however, that these coverage estimates may be affected by recall and information bias, as they relied on owner declaration in cases where vaccination cards were unavailable. Taken together, these findings underscore the need for more accessible and context-adapted vaccination strategies. Evidence from other settings shows that combining free-of-charge, door-to-door campaigns with fixed-point vaccination sites substantially improves coverage compared with fixed-point approaches alone [42,51], which currently predominate in Madagascar [52,53]. Expanding such integrated strategies could therefore play a critical role in closing existing coverage gaps among the owned dog population and advancing rabies control efforts in Antananarivo.

The estimated annual incidence of dog bites in the study area was 5 per 1,000 individuals. This aligns with findings from Moramanga, a district near the capital, where bite incidence was significantly higher, reported at 5.3 per 1,000 person-years (95% CI: 3.1–8.5) [54]. Worldwide, dog bite incidence shows substantial geographic heterogeneity, with reported rates in urban settings in Africa ranging from 0.04 to as high as 5.2 per 1,000 persons, depending on local epidemiological contexts and surveillance systems [12,35,55]. Applying this incidence to the estimated population of the Antananarivo district (approximately 3.9 million in 2023 census) [16] suggests that roughly 20,670 dog bite incidents may occur annually. In contrast, surveillance data from bite victims recorded in the same year in the Anti Rabies Centre of IPM in Antananarivo reported only 6,731 cases [56], indicating that up to two-thirds of dog bite incidents may go unreported. Although this estimate is based on a crude extrapolation and requires confirmation through more robust and longitudinal studies, it nonetheless highlights a substantial gap in accessing and/ or seeking to PEP in the capital city. Furthermore, our observed bite incidence might have been underestimated by recall bias regarding smaller bites, which could have been forgotten by the interviewed persons, while still requiring PEP. Consequently, a significant proportion of bite victims may therefore not receive life-saving treatment. This underscores the urgent need to strengthen public awareness, improve reporting mechanisms, and expand equitable access to PEP services.

A striking finding of our study is that nearly three quarters of reported bite incidents occurred in households without dogs. Although this proportion closely reflects the share of non-dog owning households in our sample, a formal comparison confirmed that bite risk did not differ significantly between household types: the relative risk of a bite incident in dog owning versus non-dog owning households was 0.75 (95% CI: 0.4–1.2, p = 0.29). This underscores the community-wide nature of rabies transmission and highlights the role of neighbourhood and free-roaming dogs in sustaining exposure risk. Similar patterns have been observed in Tanzania and the Democratic Republic of Congo [12,35],. These findings also accentuate the necessity for community-level rabies control interventions, including public education on bite prevention, promotion of responsible dog ownership, and vaccination strategies that extend beyond owned dogs to include free-roaming dogs.

Descriptive analysis of the dog bite victims revealed a predominance of men and boys among the victims (66.1%), which aligns with findings from other African contexts, such as Ethiopia [57] and the Democratic Republic of Congo [35]. However, in contrast to common global patterns [58], the majority of victims in this study (77.9%) were older than 15 years. This divergence likely reflects the local urban context; as most incidents occurred on neighbourhood streets, adults may be more frequently exposed due to work-related mobility and daily activities. It may also point to underreporting among children, who can be reluctant to disclose bites to their parents or caregivers [59]. This interpretation is strongly supported by our regression analysis, which demonstrated that households with a higher number of children under 15 years experienced a significantly increased risk of bite incidents. This association likely stems from greater exposure through playful or unsupervised interactions with dogs, coupled with the tendency for such minors-involving incidents to go unreported. Ultimately, these findings underscore that children remain a key at-risk group.

Lastly, we observed a bite peak in October, along with secondary peaks in mid- and late-year months. October marks the onset of the hot and rainy season in Madagascar [60], and higher temperatures have been associated with increased dog bite incidence [61]. Alternatively, these patterns may reflect increased human outdoor activity during school holidays and vacation periods (June-August and December-January). Such temporal trends suggest periods of heightened exposure risk, particularly for children engaging in outdoor activities. Accordingly, preventive interventions should prioritize children, especially during high-risk periods such as school holidays and the start of the school year in September. Although there is limited evidence that education alone reduces bite incidence [62], school-based interventions have consistently been shown to improve children’s knowledge and increase reporting of bite injuries [63,64], thereby strengthening post-exposure surveillance and facilitating timely access to care.

## Conclusion

This study provides a comprehensive assessment of owned dog demographics, survival, vaccination coverage, and dog bite incidence in Antananarivo, generating significant evidence that can be used as a base for the design and optimization of rabies control strategies. Our results indicate that rabies elimination among the owned dog population in Antananarivo is achievable but will require sustained, context-adapted efforts. Annual city-wide owned dog vaccination campaigns, with particular emphasis on puppies and young dogs, should be complemented by strategies to reduce roaming, improve public awareness, and expand access to timely post-exposure prophylaxis. Achieving and sustaining the 70% coverage threshold recommended by WHO nonetheless requires accounting for the ownerless dog population and for which no official local demographic data currently exist in Antananarivo. Characterizing the size, distribution, and accessibility of this population therefore constitutes a critical next step. While these recommendations are tailored to the capital, we have to keep in mind that rabies circulates throughout Madagascar [10], and comparable demographic data, for both owned and ownerless dogs, from other provinces are currently lacking. Obtaining such information is necessary to develop future strategies for dog populations in those regions to progressively move toward rabies control and elimination on a national scale and contribute to the broader goal of eliminating dog-mediated human rabies by 2030.

## Supporting information

S1 File Questionnaires. Standardized survey tool utilized during the baseline (S1) data collection phase in the Urban Commune of Antananarivo, Madagascar, 2023–2024. The file consists of three parts: (1) Household Questionnaire (socio-demographics, household composition, and economic status); (2) Dog Questionnaire (dog demography, rabies vaccination history, roaming behaviour, reproductive management, and household function); and (3) Bite Victim Questionnaire (victim characteristics, incident location, and biting animal profile).
(XLSX)

S1.1 Table Summary of participating households, dog population, and bite incidence, Urban Commune of Antananarivo, Madagascar, 2023–2024. Data are presented by Arrondissement (N = 6) and *fokontany* (N = 21).
(PDF)

S1.2 Table Summary of the censused dog population’s sex and age characteristics, Urban Commune of Antananarivo, Madagascar, 2023–2024. Data are presented by Arrondissement (N = 6) and *fokontany* (N = 21).
(PDF)

S2 Table Dog population follow-up survey in the 18 vulnerable *fokontany*, Urban Commune of Antananarivo, N = 937, 2024. Data are presented by Arrondissement (N = 6) and fokontany (N = 18).
(PDF)

## References

[1] Hampson K, Coudeville L, Lembo T, Sambo M, Kieffer A, Attlan M, et al. Estimating the global burden of endemic canine rabies. PLoS Negl Trop Dis. 2015;9(4):e0003709. doi: 10.1371/journal.pntd.0003709 25881058 PMC4400070

[2] World Health Organization. WHO Expert Consultation on Rabies: Third Report. Geneva: World Health Organization. 2018.

[3] Chevalier V, Davun H, Sorn S, Ly P, Pov V, Ly S. Large scale dog population demography, dog management and bite risk factors analysis: A crucial step towards rabies control in Cambodia. PLoS One. 2021;16(7):e0254192. doi: 10.1371/journal.pone.0254192 34237103 PMC8266089

[4] Gsell AS, Knobel DL, Kazwala RR, Vounatsou P, Zinsstag J. Domestic dog demographic structure and dynamics relevant to rabies control planning in urban areas in Africa: the case of Iringa, Tanzania. BMC Vet Res. 2012;8:236. doi: 10.1186/1746-6148-8-236 23217194 PMC3534358

[5] Morters MK, McKinley TJ, Restif O, Conlan AJK, Cleaveland S, Hampson K, et al. The demography of free-roaming dog populations and applications to disease and population control. J Appl Ecol. 2014;51(4):1096–106. doi: 10.1111/1365-2664.12279 25657481 PMC4285860

[6] Lugelo A, Hampson K, Ferguson EA, Czupryna A, Bigambo M, Duamor CT, et al. Development of Dog Vaccination Strategies to Maintain Herd Immunity against Rabies. Viruses. 2022;14(4):830. doi: 10.3390/v14040830 35458560 PMC9028497

[7] Langlois T, Hurtrez-Boussès S, de Garine-Wichatitsky M. Pet and Stray Dogs’ Contribution to Zoonotic Transmission Pathways: A Bibliometric Review. Transbound Emerg Dis. 2025;2025:5522451. doi: 10.1155/tbed/5522451 40901542 PMC12401604

[8] Diaz EW, Sila S, Raynor BB, De la Puente-León M, Levy MZ, Castillo-Neyra R. Highly dynamic population of owned dogs and implications for zoonosis control. bioRxiv. 2025. doi: 2025.04.23.650047

[9] Rajeev M, Edosoa G, Hanitriniaina C, Andriamandimby SF, Guis H, Ramiandrasoa R, et al. Healthcare utilization, provisioning of post-exposure prophylaxis, and estimation of human rabies burden in Madagascar. Vaccine. 2019;37:A35–44. doi: 10.1016/j.vaccine.2018.11.011 PMC7612383 30509692

[10] Ministère de la Santé Publique. Plan national stratégique de lutte contre la rage à Madagascar 2022–2027. Antananarivo. 2022.

[11] Andriamandimby SF, Volasoa MH, Razafindraibe NP, Ranoaritiana DB, Razafindramparany MH, Rafisandratantsoa T, et al. Rabies surveillance in Madagascar from 2011 to 2021: can we reach the target?. Front Vet Sci. 2023;10:1270532. doi: 10.3389/fvets.2023.1270532 37901098 PMC10601635

[12] Changalucha J, Steenson R, Grieve E, Cleaveland S, Lembo T, Lushasi K, et al. The need to improve access to rabies post-exposure vaccines: Lessons from Tanzania. Vaccine. 2019;37 Suppl 1(Suppl 1):A45–53. doi: 10.1016/j.vaccine.2018.08.086 30309746 PMC6863039

[13] Filla C, Rajeev M, Randriana Z, Hanitriniana C, Rafaliarison RR, Edosoa GT, et al. Lessons Learned and Paths Forward for Rabies Dog Vaccination in Madagascar: A Case Study of Pilot Vaccination Campaigns in Moramanga District. Trop Med Infect Dis. 2021;6(2):48. doi: 10.3390/tropicalmed6020048 33921499 PMC8167587

[14] Institut Pasteur de Madagascar. Rapport annuel 2024. Antananarivo, Madagascar: IPM. 2025.

[15] Ratsitorahina M, Rasambainarivo JH, Raharimanana S, Rakotonandrasana H, Andriamiarisoa MP, Rakalomanana FA. Dog ecology and demography in Antananarivo, 2007. BMC Vet Res. 2009;5:21. 10.1186/1746-6148-5-21 PMC2700795 19486516

[16] World Population Review. Madagascar cities by population 2025. 2025 [cited 2026 May 12]. Available from: https://worldpopulationreview.com/countries/madagascar-population/cities

[17] Bureau National de Gestion des Risques et des Catastrophes (BNGRC). Analyse et évaluation de la vulnérabilité urbaine « Grand Tana » (UVAA-2019). Antananarivo: Région Analamanga. 2019.

[18] Yanagisako SJ. Households in Anthropology. International Encyclopedia of the Social & Behavioral Sciences. Elsevier. 2015. p. 228–32. doi: 10.1016/b978-0-08-097086-8.12088-4

[19] Med’Vet. Rabisin. 2025 [cited 2026 May 12]. Available from: https://med-vet.fr/produits/medicament/rabisin-r

[20] Ramachandran KM, Tsokos CP. Statistical estimation. Mathematical Statistics with Applications in R. 3 ed. Academic Press. 2021. p. 179–251.

[21] Chastant-Maillard S, Reynaud K, Saint-Dizier M. Particularités de la reproduction chez la chienne. La reproduction animale et humaine. Versailles: Editions Quae. 2014. p. 751.

[22] Cole SR, Edwards JK, Naimi AI, Muñoz A. Hidden Imputations and the Kaplan-Meier Estimator. Am J Epidemiol. 2020;189(11):1408–11. doi: 10.1093/aje/kwaa086 32412079 PMC7731992

[23] Cole SR, Hudgens MG. Survival analysis in infectious disease research: describing events in time. AIDS. 2010;24(16):2423–31. doi: 10.1097/QAD.0b013e32833dd0ec 20827167 PMC2954271

[24] Lamarca R, Alonso J, Gómez G, Muñoz A. Left-truncated data with age as time scale: an alternative for survival analysis in the elderly population. J Gerontol A Biol Sci Med Sci. 1998;53(5):M337-43. doi: 10.1093/gerona/53a.5.m337 9754138

[25] CNaPS. Historique-SME. 2022 [cited 2023 September 13]. Available from: https://www.cnaps.mg/fr/historique-SME

[26] Wagenmakers EJ. How many parameters does it take to fit an elephant?. J Math Psychol. 2003;47(5):580–6.

[27] Perry BD. Dog ecology in eastern and southern Africa: implications for rabies control. Onderstepoort J Vet Res. 1993;60(4):429–36. 7777332

[28] Rajoromanana B, Nyirenda G, Edosoa GT, Andrianasolo RL, Rietmann S, Marks F, et al. Dog ecology and rabies control including canine vaccination coverage: Impacts from a survey in Madagascar. PLoS One. 2024;19(5):e0302690. doi: 10.1371/journal.pone.0302690 38722982 PMC11081359

[29] Flockhart DTT, Rowan AN, Boone JD. Owned dog population size and ownership patterns in Costa Rica. Front Vet Sci. 2022;9:946603. doi: 10.3389/fvets.2022.946603 36532345 PMC9751483

[30] World Health Organization, Food and Agriculture Organization of the United Nations, World Organisation for Animal Health, Global Alliance for Rabies Control. Zero by 30: the global strategic plan to end human deaths from dog-mediated rabies by 2030. Geneva: WHO. 2015.

[31] Murungi MK, Thomas LF, Bor N, Masaku I, Anyango M, Munywoki PN, et al. Demography of owned dogs across an East African continuum of high-low human density. Prev Vet Med. 2025;239:106518. doi: 10.1016/j.prevetmed.2025.106518 40158244

[32] Tenzin T, Hikufe EH, Hedimbi N, Athingo R, Shikongo MB, Shuro T, et al. Dog ecology and rabies knowledge, attitude and practice (KAP) in the Northern Communal Areas of Namibia. PLoS Negl Trop Dis. 2024;18(2):e0011631. doi: 10.1371/journal.pntd.0011631 38315727 PMC10881021

[33] Hergert M, Le Roux K, Nel LH. Characteristics of owned dogs in rabies endemic KwaZulu-Natal province, South Africa. BMC Vet Res. 2018;14(1):278. doi: 10.1186/s12917-018-1604-z 30200962 PMC6131809

[34] Mindekem R, Kayali U, Yemadji N, Ndoutamia AG, Zinsstag J. Impact of canine demography on rabies transmission in N’djamena, Chad. Med Trop (Mars). 2005;65(1):53–8. 15903078

[35] Mbilo C, Kabongo J-B, Pyana PP, Nlonda L, Nzita RW, Luntadila B, et al. Dog Ecology, Bite Incidence, and Disease Awareness: A Cross-Sectional Survey among a Rabies-Affected Community in the Democratic Republic of the Congo. Vaccines (Basel). 2019;7(3):98. doi: 10.3390/vaccines7030098 31454908 PMC6789516

[36] Wallace RM, Mehal J, Nakazawa Y, Recuenco S, Bakamutumaho B, Osinubi M, et al. The impact of poverty on dog ownership and access to canine rabies vaccination: results from a knowledge, attitudes and practices survey, Uganda 2013. Infect Dis Poverty. 2017;6(1):97. doi: 10.1186/s40249-017-0306-2 28569185 PMC5452361

[37] Davlin SL, Vonville HM. Canine rabies vaccination and domestic dog population characteristics in the developing world: a systematic review. Vaccine. 2012;30(24):3492–502. doi: 10.1016/j.vaccine.2012.03.069 22480924

[38] Acosta-Jamett G, Cleaveland S, Cunningham AA, Bronsvoort BMD. Demography of domestic dogs in rural and urban areas of the Coquimbo region of Chile and implications for disease transmission. Prev Vet Med. 2010;94(3–4):272–81. doi: 10.1016/j.prevetmed.2010.01.002 20096943

[39] Kitala P, McDermott J, Kyule M, Gathuma J, Perry B, Wandeler A. Dog ecology and demography information to support the planning of rabies control in Machakos District, Kenya. Acta Trop. 2001;78(3):217–30. doi: 10.1016/s0001-706x(01)00082-1 11311185

[40] Belsare AV, Gompper ME. Assessing demographic and epidemiologic parameters of rural dog populations in India during mass vaccination campaigns. Prev Vet Med. 2013;111(1–2):139–46. doi: 10.1016/j.prevetmed.2013.04.003 23664490

[41] Chidumayo NN. System dynamics modelling approach to explore the effect of dog demography on rabies vaccination coverage in Africa. PLoS One. 2018;13(10):e0205884. doi: 10.1371/journal.pone.0205884 30359399 PMC6201891

[42] Barbosa Costa G, Ludder F, Monroe B, Dilius P, Crowdis K, Blanton JD, et al. Barriers to attendance of canine rabies vaccination campaigns in Haiti, 2017. Transbound Emerg Dis. 2020;67(6):2679–91. doi: 10.1111/tbed.13622 32438530 PMC7754310

[43] Wiethoff JP, Omar KN, Othman OJ, Moh’d AZ, Suleiman TS, Mlenge MH, et al. Situation analysis and prospects for One Health implementation in Zanzibar, Tanzania. One Health. 2025;21:101198. doi: 10.1016/j.onehlt.2025.101198 41017845 PMC12466235

[44] Aréchiga Ceballos N, Puebla Rodríguez P, Aguilar Setién Á. The New Face of Human Rabies in Mexico, What’s Next After Eradicating Rabies in Dogs. Vector Borne Zoonotic Dis. 2022;22(2):69–75. doi: 10.1089/vbz.2021.0051 35175137

[45] Auerswald H, Guillebaud J, Durand B, Le Vu M, Sorn S, In S, et al. Bayesian modeling of post-vaccination serological data suggests that yearly vaccination of dog aged <2 years old is efficient to stop rabies circulation in Cambodia. PLoS Negl Trop Dis. 2024;18(4):e0012089. doi: 10.1371/journal.pntd.0012089 38635851 PMC11060556

[46] Arief RA, Hampson K, Jatikusumah A, Widyastuti MDW, Sunandar, Basri C, et al. Determinants of Vaccination Coverage and Consequences for Rabies Control in Bali, Indonesia. Front Vet Sci. 2017;3:123. doi: 10.3389/fvets.2016.00123 28119919 PMC5220097

[47] Wallace RM, Cliquet F, Fehlner-Gardiner C, Fooks AR, Sabeta CT, Setién AA, et al. Role of Oral Rabies Vaccines in the Elimination of Dog-Mediated Human Rabies Deaths. Emerg Infect Dis. 2020;26(12):1–9. doi: 10.3201/eid2612.201266 33219786 PMC7706920

[48] Knobel DL, Laurenson MK, Kazwala RR, Boden LA, Cleaveland S. A cross-sectional study of factors associated with dog ownership in Tanzania. BMC Vet Res. 2008;4:5. doi: 10.1186/1746-6148-4-5 18230137 PMC2262882

[49] Enders-Slegers M-J, Hediger K. Pet Ownership and Human–Animal Interaction in an Aging Population: Rewards and Challenges. Anthrozoös. 2019;32(2):255–65. doi: 10.1080/08927936.2019.1569907

[50] Dreyfus A, Volasoa MH, Guis H, Razafindraibe NP, Razafindramparany MH, Arivony NL, et al. Challenges of rabies surveillance in Madagascar based on a mixed method survey amongst veterinary health officers. Front Vet Sci. 2024;11:1270547. doi: 10.3389/fvets.2024.1270547 38487708 PMC10938849

[51] Sánchez-Soriano C, Gibson AD, Gamble L, Burdon Bailey JL, Green S, Green M, et al. Development of a high number, high coverage dog rabies vaccination programme in Sri Lanka. BMC Infect Dis. 2019;19(1):977. doi: 10.1186/s12879-019-4585-z 31747889 PMC6868729

[52] Global Alliance for Rabies Control. Campagne de vaccination antirabique. 2024 [cited 2025 June 12]. Available from: https://rabiesalliance.org/world-rabies-day/event/campagne-de-vaccination-antirabique

[53] SEGA One Health Network. Supporting Madagascar in the fight against rabies. 2024 [cited 2025 June 12]. Available from: https://segaonehealth.org/en/supporting-madagascar-rabies/

[54] Leblanc C, Kassié D, Ranaivoharimina M, Rakotomanana EFN, Mangahasimbola RT, Randrianarijaona A, et al. Mixed methods to evaluate knowledge, attitudes and practices (KAP) towards rabies in central and remote communities of Moramanga district, Madagascar. PLoS Negl Trop Dis. 2024;18(3):e0012064. doi: 10.1371/journal.pntd.0012064 38551968 PMC11006160

[55] Gborie SR, Issahaku GR, Bonful HA, Bandoh DA, Squire J, Ameme DK, et al. Analysis of dog bite surveillance data, Volta Region, Ghana, 2020. Front Trop Dis. 2023;4:1164917.

[56] Institut Pasteur de Madagascar. Rapport annuel 2023. Antananarivo, Madagascar: IPM. 2024.

[57] Regassa BT, Tosisa W, Eshetu D, Mulu A, Hundie GB. Incidence, risk factors, and control of Rabies in Ethiopia: A systematic review and meta-analysis. PLoS Negl Trop Dis. 2025;19(3):e0012874. doi: 10.1371/journal.pntd.0012874 40106430 PMC11922250

[58] World Health Organization. Rabies fact sheets. World Health Organization. 2024 [cit4ed 2026 May 12]. Available from: https://www.who.int/news-room/fact-sheets/detail/rabies

[59] Shen J, Rouse J, Godbole M, Wells HL, Boppana S, Schwebel DC. Systematic Review: Interventions to Educate Children About Dog Safety and Prevent Pediatric Dog-Bite Injuries: A Meta-Analytic Review. J Pediatr Psychol. 2017;42(7):779–91. doi: 10.1093/jpepsy/jsv164 26773009 PMC5896610

[60] Tadross M, Randriamarolaza LYA, Rabefitia Z, Yip Z. Climate change in Madagascar : recent past and future. Antananarivo: Meteo Madagascar. 2008.

[61] Ahmed T. A short report on epidemiological investigation of dog bite cases in association with temperature rise as a part of climate change. J Public Health Epidemiol. 2019;11(1):1–5.

[62] Auplish A, Clarke AS, Van Zanten T, Abel K, Tham C, Bhutia TN, et al. Estimating the intra-cluster correlation coefficient for evaluating an educational intervention program to improve rabies awareness and dog bite prevention among children in Sikkim, India: A pilot study. Acta Trop. 2017;169:62–8. doi: 10.1016/j.actatropica.2016.12.032 28040481

[63] Burdon Bailey JL, Gamble L, Gibson AD, Bronsvoort BMC, Handel IG, Mellanby RJ, et al. A rabies lesson improves rabies knowledge amongst primary school children in Zomba, Malawi. PLoS Negl Trop Dis. 2018;12(3):e0006293. doi: 10.1371/journal.pntd.0006293 29522517 PMC5862537

[64] Deray R, Rivera C, Gripon S, Ulanday C, Roces MC, Amparo AC, et al. Protecting children from rabies with education and pre-exposure prophylaxis: A school-based campaign in El Nido, Palawan, Philippines. PLoS One. 2018;13(1):e0189596. doi: 10.1371/journal.pone.0189596 29293571 PMC5749686

