# Supplementary material for: Owned dog population profile, dog ownership and bite risk determinants: Key steps to rabies control in Antananarivo, Madagascar
Source: PLoS One. 2026 Oct 16;21(10):e0360417. doi: 10.1371/journal.pone.0360417 (PMC13674757; doi:10.1371/journal.pone.0360417)
Supplement: Supplementary file 1 [file pone.0360417.s002.pdf]

S1.1 Table. Summary of participating households, dog population, and bite incidence, Urban Commune of Antananarivo, Madagascar, 2023–2024.

Arrondissement	*Fokontany*	Total population	Total Household	Participating Household	Total participating household member	Participating Household with dog	Total censed dog	Dog human ratio	Dog bite recorded 2023 – 2024
**(N=6)**	**(N=21)**			**(N=2,518)**	**(N=11,150)**	**(N=767)**	**(N=1,273)**	**(1 :9)**	**(N=90)**
1	67 ha	14,323	2,903	111 (4.4%)	497	22(4.4%)	41	1 :12	2
1	Ambohimiandra*	8123	3457	102(4.0%)	434	26(6.0%)	43	1 :10	0
1	Ankadivato*	NA	NA	106(4.2%)	427	20(4.7%)	27	1 :16	0
1	Fiata	5,145	NA	122(4.8%)	532	27(5.1%)	40	1 :13	9
1	Isotry	6 ,135	1,373	134(5.3%)	614	47(7.7%)	69	1 :09	9
2	Ambanidia	9,321	1,714	124(4.9%)	542	31(5.7%)	40	1 :13	5
2	Ambohipo	31,253	9,350	116(4.6%)	477	19(4.0%)	37	1 :13	5
2	Mahazoarivo	10,321	NA	119(4.7%)	574	15(2.6%)	37	1 :15	4
3	Ampandrana	5,450	1,450	102(4.0%)	473	37(7.8%)	54	1 :09	1
3	Ankazomanga Andraharo	3,543	NA	115(4.6%)	520	43(8.8%)	72	1 :07	7
3	Mahavoky	4,239	1,184	117(4.6%)	552	32(5.8%)	52	1 :10	9
4	Anosibe	14,040	3,910	107(4.2%)	485	32(6.6%)	63	1 :7.7	5
4	Namontana	27,964	5,610	106(4.2%)	461	30(6.5%)	66	1 :07	4
4	Tsimbazaza	20,023	3593	117(4.6%)	510	37(7.2%)	55	1 :09	10
5	Ambatomaro	30,223	7,647	132(5.3%)	586	53(9.1%)	93	1 :06	4
5	Analamahitsy Tanana	15,374	NA	128(5.1%)	524	28(5.3%)	40	1 :13	2
5	Ivandry*	NA	NA	124(4.9%)	550	42(7.6%)	82	1 :07	0
5	Soavimasoandro	32,690	10,110	126(5.0%)	552	62(11.3%)	97	1 :06	5
6	Ambohimitsinjo	3,549	1,070	134 (5.3%)	595	68(11.4%)	120	1 :05	1
6	Antanety Atsimo	7,259	2,427	127(5.04%)	604	33(5.4%)	51	1 :12	6
6	Antanjombe	4,320	1,168	149(5.92%)	641	63 (9.8%)	94	1 :07	2

Data are presented by arrondissement (N = 6) and fokontany (N = 21)

*Economically affluent fokontany
