# Supplementary material for: Owned dog population profile, dog ownership and bite risk determinants: Key steps to rabies control in Antananarivo, Madagascar
Source: PLoS One. 2026 Oct 16;21(10):e0360417. doi: 10.1371/journal.pone.0360417 (PMC13674757; doi:10.1371/journal.pone.0360417)
Supplement: Supplementary file 2 [file pone.0360417.s003.pdf]

S1.2 Table. Summary of the censused dog population's sex and age characteristics, Urban Commune of Antananarivo, Madagascar, 2023–2024.

Arrondissement	*Fokontany*	Total censed	Male dog	Female dog	Male to female ratio	Median age	Mean age	Median age variation according to the fokontany *P-value*
**(*N*=6)**	***(N=21)***	**(*N*=1,273)**	**63.5% (809/1,273)**	**36.4% (464/1,273)**	**(1 :0.5)**	**(3 yrs)**	**(4yrs)**	
1	67 ha	41	14	27	1 :1.9	3.0	3.1	0.88
1	Ambohimiandra*	43	24	19	1 :0.7	4.0	4.6	0.01
1	Ankadivato*	27	17	10	1 :0.5	3.0	4.9	0.04
1	Fiata	40	22	18	1 :0.8	1.5	3.0	0.75
1	Isotry	69	41	28	1 :0.7	2.0	2.9	0.39
2	Ambanidia	40	26	13	1 :0.5	3.0	3.7	0.38
2	Ambohipo	37	19	18	1 :0.9	2.0	2.6	0.31
2	Mahazoarivo	37	23	14	1 :0.6	2.0	2.5	0.12
3	Ampandrana	54	23	31	1 :1.3	3.0	3.4	0.51
3	Ankazomanga Andraharo	72	45	27	1 :0.6	2.0	2.9	0.57
3	Mahavoky	52	28	24	1 :0.8	3.0	3.6	0.33
4	Anosibe	63	47	17	1 :0.3	2.0	2.5	0.06
4	Namontana	66	36	30	1 :0.8	2.0	2.1	0.00
4	Tsimbazaza	55	39	16	1 :0.4	3.0	3.7	0.23
5	Ambatomaro	93	69	24	1 :0.3	2.0	3.2	0.91
5	Analamahitsy Tanana	40	23	17	1 :0.7	2.0	2.5	0.10
5	Ivandry*	82	50	32	1 :0.6	3.0	3.7	0.14
5	Soavimasoandro	97	70	26	1 :0.3	3.0	3.4	0.45
6	Ambohimitsinjo	120	87	33	1 :0.3	3.0	3.1	0.91
6	Antanety Atsimo	51	36	15	1 :0.4	2.0	2.0	0.00
6	Antanjombe	94	70	25	1 :0.3	3.0	3.4	0.50

Data are presented by arrondissement (N = 6) and fokontany (N = 21)

*Economically affluent fokontany
