# Supplementary material for: Owned dog population profile, dog ownership and bite risk determinants: Key steps to rabies control in Antananarivo, Madagascar
Source: PLoS One. 2026 Oct 16;21(10):e0360417. doi: 10.1371/journal.pone.0360417 (PMC13674757; doi:10.1371/journal.pone.0360417)
Supplement: Supplementary file 3 [file pone.0360417.s004.pdf]

S2 Table. Dog population follow-up survey in the 18 vulnerable fokontany, Urban Commune of Antananarivo, N= 937, 2024.

Arrondissement (N=6)	*Fokontany* (N=18)	Total censed (N=1,273)	Followed dogs (N=937)	% of followed dog	Male (N=603)	Female (N=334)	Nbr of dead male (N=64)	Nbr of dead female (N=34)	Mean age of dead male	Mean age of dead female	Dog still alive (N=765)	Dog censored (N=74)
1	67_ha	41	28	68.3	11	17	0	0	NA	NA	21	7
1	Fiata	40	27	67.5	15	12	3	2	3.9	6.0	21	1
1	Isotry	69	63	91.3	35	28	4	5	3.7	3.6	51	3
2	Ambanidia	40	36	90.0	23	13	4	1	3.9	0.1	30	1
2	Ambohipo	37	30	81.1	18	12	3	2	7.1	3.1	19	6
2	mahazoarivo	37	31	83.7	18	13	1	3	2.0	3.3	26	1
3	Ampandrana	54	46	85.2	20	26	1	2	7.0	8.5	36	7
3	Ankazomanga_Andraharo	72	66	91.7	41	25	7	2	4.1	3.5	46	11
3	Mahavoky	52	46	88.5	24	22	1	1	5.0	1.0	44	0
4	Anosibe	63	56	88.9	41	15	3	1	4.1	1.0	45	7
4	Namontana	66	65	98.5	36	29	4	3	1.8	0.1	50	8
4	Tsimbazaza	55	48	87.3	34	14	3	2	6.6	6.5	41	2
5	Ambatomaro	93	76	81.7	56	20	7	6	4.3	1.5	61	2
5	Analamahitsy_Tanana	40	31	77.5	18	13	1	1	0.3	3.0	25	4
5	Soavimasoandro	97	89	91.7	66	23	8	1	4.7	5.0	75	5
6	Ambohimitsinjo	120	89	74.2	66	23	5	1	3.8	7.0	78	5
6	Antanety_Atsimo	51	40	78.4	29	11	4	0	1.9	NA	34	2
6	Antanjombe	94	70	74.5	52	18	5	1	3.9	2.0	62	2

Data are presented by arrondissement (N = 6) and fokontany (N = 18)
